# Folic Acid Absorption Characteristics and Effect on Cecal Microbiota of Laying Hens

**DOI:** 10.3389/fvets.2021.720851

**Published:** 2021-08-17

**Authors:** Yan Bai, Rui Wang, Yu Yang, Ruirui Li, Xiaotian Wu

**Affiliations:** ^1^Laboratory of Poultry Production, College of Animal Science, Shanxi Agricultural University, Jinzhong, China; ^2^Department of Life Sciences, Luliang University, Luliang, China

**Keywords:** laying hen, folic acid, absorption, gut microbiota, functional prediction

## Abstract

This experiment was conducted to investigate the characteristics of folic acid (FA) absorption in laying hens and the effect of FA supplementation on cecal microbiota. A total of 432 healthy hens (30-week-old) were randomly assigned to four diets supplemented with FA: 0, 1, 6, and 24 mg/kg of feed for 8 w. Blood, duodenum, jejunum, ileum, cecum, and cecal chyme samples (six samples per treatment) were collected from the hens at the end of the feeding trial. Expression profiles of folate transport and transformation genes in intestine and cecal microbiota were detected. Results showed that serum folate level significantly increased (*P* < 0.01) with an increase in dietary FA supplementation, reaching a plateau at 6 mg/kg FA supplementation. The expression of FA transport and transformation genes was not affected in the cecum (*P* > 0.05) by dietary FA supplementation; however, it was affected in the duodenum, jejunum, and ileum and mostly showed a downward trend in treatment groups (*P* < 0.05). The genes affected include duodenal folate receptor (*Folr*) and dihydrofolate reductase (*Dhfr*), jejunal proton-coupled folate transporter (*Pcft*) and reduced folate carrier (*Rfc*), and ileal ATP binding cassette subfamily C member (*Abcc2*), *Abcc3, Rfc, Folr*, and *Dhfr*. Furthermore, according to the operational taxonomic unit classification and taxonomic position identification, the cecal microbiota population of the hens was not affected by dietary FA supplementation at the phylum, class, order, family, genus, and species levels (*P* > 0.05). However, the relative abundance of some microbiota was affected by dietary FA supplementation (*P* < 0.05). In conclusion, FA transport from the intestinal lumen into enterocytes, and then into the bloodstream, is strictly regulated, which may be associated with the regulation of the expression profiles of genes involved in FA absorption. Pathogenic bacteria decreased in the cecum, especially at 24 mg/kg supplementation, but the beneficial bacteria (Bifidobacteriaceae) decreased at this level, too. Overall, FA supplementation at 6 mg/kg, which was selected for folate-enriched egg production, did not affect the health and metabolism of laying hens negatively.

## Introduction

The term folate (vitamin B9) refers to a group of compounds belonging to the water-soluble vitamins, playing an important role in one-carbon metabolism (1). Deficiency in folate has been linked to a variety of disorders, including anemia (2), neural tube defects during fetal development (3), mental illness (4), cancers (5), eye diseases (6). People have gradually realized folate's importance and have started looking for ways to supplement folate. Humans and animals lack the enzymes to synthesize folate *de novo*, so they must obtain it from their food. Green vegetables, yeast, animal livers, egg yolk, folic acid (FA)-fortified foods, or additives are all good folate sources for humans (7, 8). Because of folate's instability in vegetable sources (9, 10), the potential negative effects of synthetic FA (11–13), egg yolk folate draws considerable attention thanks to its stability, natural occurrence, and high bioavailability (14, 15). Studies have shown that folate content in egg yolk can be increased by supplementing laying hen feed with FA. However, attempts to increase folate concentration in eggs beyond the achieved enrichment level were unsuccessful. Egg folate has reached a maximum plateau and does not increase further with increased dietary FA (16–19). Intestinal absorption of FA is believed to be one of the main factors affecting the transfer of dietary FA into the egg.

The folate sources for laying hens are the feedstuff and synthetic crystalline FA. Plant-derived folate is mostly in the form of polyglutamate with 5–9 glutamate tails (20), and it is absorbed in the intestinal tract. While FA can be absorbed directly, the polyglutamated forms must be first converted to their monoglutamate form by folate hydrolase (1). Within the chicken intestinal tract, the monoglutamate forms are transported across the enterocytes through the action of specific transporters: folate receptor (FOLR), reduced folate carrier (RFC) (21), and proton-coupled folate transporter (PCFT) (22). Once inside the enterocytes, synthetic FA is reduced to tetrahydrofolate (THF) by dihydrofolate reductase (DHFR) to gain a metabolic activity similar to other folate species. Multidrug-resistance-associated protein 2 (MRP2), coded by ATP binding cassette subfamily C member 2 (*ABCC2*), is expressed at the small intestine apical brush-border membrane and can pump folates back into the intestinal lumen, thereby countering the inward transport mediated by PCFT. MRP3 (coded by *ABCC3*) and MRP5 (coded by *ABCC5*) are expressed in the basolateral membrane where they export folate for transit into the vascular system (23). FA absorption rate in the everted intestinal sac model of laying hens reaches a plateau after a certain FA content (24). The regulation of FA intestinal intake is crucial for folate transport to the egg, but the specific mechanism is unclear. The absorption transporter might be regulated at the gene expression, protein and translation levels. Alternatively, the process of enterocyte FA excretion is regulated, thus saturating FA absorption in the intestinal epithelial cells. In this study, the mRNA expression levels of *Pcft, Rfc, Abcc2, Abcc3, Abcc5, Folr*, and *Dhfr* in the duodenum, jejunum, ileum, and cecum were detected to understand folic acid absorption characteristics in the intestinal tract of laying hens.

Owing to the specific and saturable process of folate transport into the enterocytes (25), a large amount of FA or its degradants may be excreted through feces. When a large amount of FA is fed to hens for the production of folate-enriched eggs, excess FA is transferred into the cecum. The cecum contains several microbes, which play an important role in maintaining the nutritional status of birds (26, 27). Some of the microbes present in the cecum are capable of synthesizing folate, whereas others depend on dietary folate for growth. Gut microbe-synthesized folate is an important source for the host animal (28). The gut microbiota is an important factor regulating gastrointestinal tract homeostasis and energy metabolism, as well as the immune and central nervous systems of laying hens. Chicken performance and gut health dependent on the complex gut microbial community, which plays a role in nutrient assimilation, biosynthesis, and prevention of pathogen colonization.

Several studies have examined the gut microbiota of chickens under various experimental conditions; however, studies on the effect of FA on the intestinal microbiota of poultry are limited. Therefore, the aim of this study was to assess the effects of FA on the gut microbiota of laying hens and to determine whether the ecological balance of the cecal microorganisms is affected by FA supplementation. Additionally, we investigated the effect of FA supplementation on the cecal microbiota structure of laying hens using 16S rRNA high-throughput sequencing.

## Methods and Materials

The study was carried out in accordance with the Chinese guidelines for animal welfare and experimental protocol, and was approved by Shanxi Agricultural University Animal Experiment Ethics Committee (SXAU-EAW-2018-002Chi.001).

### Animals, Diets, and Experimental Design

The 8-week experiment (2-week pre-experiment and 6-week formal experiment) was performed in Xingmin Animal Husbandry Industry Cooperative (Taigu, Jinzhong, Shanxi, China) and a single factor test design was adopted. Four hundred and thirty-two 30-week-old healthy Jinghong laying hens were randomly allotted into four groups, each with six replicates of 18 birds. The replicates were divided into six cages with three birds per cage [50 × 50 × 45/38 cm (long × wide × front high/back high)]. The management of the indoor light, temperature followed the chickens' breeding manual (16 h:8 h light: dark cycles, 20 ± 3°C). The chickens had free access to water and food. The dietary treatments consisted of a corn-soybean meal-based diet (Table 1) supplemented with folic acid at 0, 1, 6, and 24 mg/kg (marked as FA0, FA1, FA6, and FA24, respectively), which was purchased from Ketai Biological Co. Ltd. (Shijiazhuang, Hebei, China) with purity of 99.8%. FA0 acted as the control group, 1 mg/kg was the supplemental amount for laying hens according the breeding manual, 6 mg/kg was the most appropriate dose for folate-enriched eggs production based on our previous results, and 24 mg/kg for excess added treatment. The production performance and egg quality measurements were performed weekly and detailed in Appendix Table 1.

**Table 1:** Ingredients and Composition (g/kg diet) of the basal diet (air-dry basis).

**Ingredients**	**Content**	**Nutrient levels^c^**	**Nutrient Composition**
Corn	645	Crude protein	164.6
Soybean meal	210	Crude fiber	30.0
Cottonseed meal	10	Ether extract	28.9
Linseed meal	20	Ash	126.1
Limestone	96.6	Calcium	35.1
NaCl	3	Total phosphorus	5.0
*DL*-Methionine (98%)	1.4	Available phosphorus	2.2
L-Lysine·H_2_SO_4_ (70%)	0.4	Methionine	3.9
Choline Chloride (50%)	1	Lysine	7.8
Vitamin premix^a^	0.4	Threonine	5.8
Minerals premix^b^	2	Folate (mg /kg)	0.35
Calcium hydrophosphate	10	Metabolism energy /(MJ/kg)	11.08
Phytase (5000 IU/g)	0.2		
Total	1,000		

a *The multivitamin premix provided per kilogram of diet: vitamin A 14,400 IU, vitamin D 5,400 IU, vitamin K3 3.2 mg, vitamin B1 2.4 mg, vitamin B2 10 mg, vitamin B12 0.025 mg, vitamin E 32 mg, biotin 0.16 mg, pantothenic acid 14 mg, and niacin 48 mg*.

b *The mineral premix provided per kilogram of diet: Cu (as copper sulfate) 8 mg, Fe (as ferrous sulfate) 50 mg, Mn (as manganese sulfate) 100 mg, Zn (as zinc sulfate) 90 mg, I (as potassium iodide) 0.40 mg, Se (as sodium selenite) 0.36 mg, and Co (as cobalt sulfate) 0.26mg*.

c *The composition of ME, available phosphorus, amino acids were calculated, the others were measured*.

### Sample Collection

At the end of the experiment, one hen with a final body weight (BW) close to mean BW of each replication (a total of six samples per treatment) was euthanized and subjected to a full post-mortem examination following an overnight fast. Immediately before euthanasia, blood was collected from the wing vein into 5-mL vacuum tubes without anticoagulant to obtain a serum sample. The duodenum, jejunum, ileum, cecum, and the cecal chyme were sampled aseptically.

Blood samples were incubated in a water bath at 37°C for 1 h, followed by centrifugation at 3000 rpm for 10 min (TG16-WS, Xiangli Scientific Instruments Co. Ltd. Changsha, Hunan, China). The upper serum layer was aspirated with a pipette into a 0.5-mL Eppendorf tubes and immediately preserved at −20°C, pending further analysis. About 2 cm of the anterior duodenum, the middle jejunum, and ileum, and the right cecum were rinsed in precooled saline, packed into separate cryogenic tubes, plunged into liquid nitrogen, and then maintained at −80°C, pending detection by quantitative real-time polymerase chain reaction (qRT-PCR). The contents of the left cecum were collected for 16S rRNA sequencing to identify the gut microbiota.

### Folate and Homocysteine Content in the Serum

The folate and homocysteine serum levels were assayed using a chicken enzyme linked immunosorbent assay kit (Solarbio Science and Technology Ltd., Beijing, China) following the manufacturer's instructions.

### Quantitative Real-Time PCR Analysis of Gene Expression

The intestinal samples were ground with liquid nitrogen, and their total RNA was extracted using the RNAiso Plus reagent (Takara Biotechnology Co., Ltd, Dalian, Liaoning, China) following the manufacturer's instructions. The quantity and quality of the extracted total RNA were determined with a spectrophotometer (NanoDrop 2000, Thermo Fisher Scientific, Waltham, MA, USA) at 260 and 280 nm. First-strand cDNA was synthesized from 1,000 ng of total RNA using a PrimeScriptTM RT reagent Kit with gDNA Eraser (Perfect Real Time; Takara Biotechnology Co., Ltd.) following the manufacturer's instructions. The genes' mRNA level (gene names and the primer sequences were listed in Table 2) was analyzed by quantitative real-time PCR. The genes expression was quantified in triplicate with a StepOnePlus Real-Time PCR System (Applied Biosystems, Foster City, CA, USA) and TB Green Premix Ex Taq II (Tli RNase H Plus; Takara Biotechnology Co., Ltd.) following the manufacturer's instructions, at a reaction volume of 20 μl. The relative expression of the target genes was calculated using the 2^−ΔΔCt^ method, with β-actin as the housekeeping gene.

**Table 2:** Nucleotide sequences of the primers used in the qRT-PCR assay.

**Gene**	**Primer sequence (5^**′**^-3^**′**^)**	**GenBank accession no**.	**Size (bp)**
*Abcc2*	F: CTACGGACATCAGCAGCGGTTC	XM_015288821.2	133
	R: GACCACCAGGCTCCCAACAAAC		
*Abcc3*	F: GCTGTTCGGTGTCCTGCTGTG	XM_015295526.2	87
	R: GGTGGTGGTGTTCTGCTCTGC		
*Abcc5*	F: AAGACCTGACCTAGCCATCCTTCC	XM_004943392.3	89
	R: TTCTCTGACGCTGTCCTCCACTC		
*Pcft*	F: TGGTCGGTGAGACGGAGCAG	NM_001205066.1	153
	R: AAGGTGGAAGGGTAGAGGGAGTTG		
*Rfc*	F: CGCTGGTGATTGGAGTGGTGAC	NM_001006513.1	103
	R: GGTAGGAGCCACGGAAGAGGAC		
*Folr*	F: CAAAGAGGACTGCGAGGAATGGTG	NM_204834.1	87
	R: GTTGCCCAGTTCCAGCCCTTG		
*Dhfr*	F: GTCAGAACATGGGCATCGGGAAG	XM_025144726.1	91
	R: GAGGTGCTGGTCATTCTCTGGAAG		
*β-actin*	F: CAACACAGTGCTGTCTGGTGGTA	NM_205518.1	205
	R: ATCGTACTCCTGCTTGCTGATCC		

*Abcc, ATP-binding cassette subfamily C member; Dhfr, dihydrofolate reductase; F, forward primer; Folr, folate receptor; Pcft, proton coupled folate transporter; R, reverse primer; Rfc, reduced folate carrier*.

### Microbial DNA Extraction, 16S rRNA Gene Amplification of the V3-V4 Region, Sequencing, and Bioinformatic Analysis

Total genomic DNA of cecal chyme microbiota were extracted using the Fast DNA SPIN extraction kits (MP 122 Biomedicals, Santa Ana, CA, USA), following the manufacturer's instructions, and stored at −20°C, pending further analysis. The quantity and quality of the extracted DNAs were assessed using NanoDrop ND-1000 spectrophotometer (Thermo Fisher Scientific, Waltham, MA, USA) and agarose gel electrophoresis, respectively.

PCR amplification of the bacterial 16S rRNA genes in the V3-V4 region was performed using the 338F forward primer (5′-ACTCCTACGGGAGGCAGCA-3′) and the 806R reverse primer (5′-GGACTACHVGGGTWTCTAAT-3′). Sequencing was performed using the Illumina MiSeq platform with MiSeq Reagent Kit v3 at the Shanghai Personal Biotechnology Co., Ltd (Shanghai, China).

The Quantitative Insights into Microbial Ecology (QIIME, v1.8.0) pipeline was employed to process the sequencing data, as previously described (29). Briefly, raw sequencing reads with exact matches to the barcodes were assigned to respective samples and identified as valid sequences. The low-quality sequences were filtered based on the following criteria (30, 31): sequences that had a length of < 150 bp or an average Phred score of < 20, contained ambiguous bases or mononucleotide repeats of > 8 bp. Paired-end reads were assembled using Fast Length Adjustment of Short reads (FLASH) (32). After chimera detection, the remaining high-quality sequences were clustered into operational taxonomic units (OTUs) at 97% sequence identity by UCLUST (33). A representative sequence was selected from each OTU using default parameters. OTU taxonomic classification was done by Basic Local Alignment Search Tool (BLAST), searching the Greengenes Database (34) against sequence sets, using the best hit (35). An OTU table was generated to record the abundance of each OTU in each sample and the taxonomy of these OTUs. OTUs containing <0.001% of total sequences across all samples were discarded. To minimize differences in sequencing depth across samples, an averaged, rounded rarefied OTU table was generated by averaging 100 evenly resampled OTU subsets under 90% of the minimum sequencing depth for further analysis.

Sequence data analyses were mainly performed using QIIME and packages in R (Version 3.2.0). OTU-level α diversity indices, such as Chao1 richness estimator, Abundance-based Coverage Estimator (ACE) metric, Shannon diversity index, and Simpson index, were calculated using the OTU table in QIIME. Kruskal–Wallis test by ranks was applied to compare the relative abundance of bacteria at the phylum, family, and genus levels. Spearman correlation coefficient was used to analyze the correlation between FA supplementation and bacterial abundance.

Microbial functions were predicted by Phylogenetic Investigation of Communities by Reconstruction of Unobserved States (PICRUSt) based on high-quality sequences, and mapped the data to Kyoto Encyclopedia of Genes and Genomes (KEGG) pathway database. The correlation values between the relative abundance of KEGG pathways and the bacteria were shown in a heatmap based on spearman correlation analysis.

### Statistical Analysis

The data of serum parameters, and expression level of genes were analyzed by one-way analysis of variance (ANOVA) with the Duncan *post hoc* test for multiple comparisons, using IBM SPSS statistical software for Windows (Version 26.0, IBM Corp., Armonk, NT, USA). Results are expressed as treatment means with their pooled standard error of the mean. A probability value of *P* < 0.05 was considered statistically significant.

## Results

### Serum Folate and Homocysteine Concentration

Serum folate and homocysteine levels were measured to determine the effect of dietary FA supplementation on folate metabolism in hens. There was an increase (*P* < 0.001) in serum folate level and a decrease (*P* = 0.001) in serum homocysteine level with increase in dietary FA supplementation (Table 3). Moreover, serum folate level was negatively correlated (*R* = −0.794; *P* = 0.002) with serum homocysteine level.

**Table 3:** Effect of folic acid supplementation on serum folate and homocysteine levels.

**Items**	**FA0**	**FA1**	**FA6**	**FA24**	**SEM**	***P*-value**
Folate (ng/L)	226.3^Aa^	241.0^Aa^	285.6^Bb^	285.4^Bb^	8.3	< 0.001
Homocysteine (μmol/L)	13.11^Aa^	11.81^Aa^	6.85^Bb^	6.94^Bb^	0.80	0.001

*Data are expressed as mean and pooled standard error of the mean (SEM) (n = 6). Means values within a row with different lowercase superscript letters are significantly different at P < 0.05, and mean values with different uppercase superscript letters are significantly different at P < 0.01. FA = folic acid supplementation. The numbers after FA refer to the amounts added in mg/kg feed*.

### Relative mRNA Expression of Folic Acid Transfer Genes in the Duodenum, Jejunum, Ileum, and Cecum

The cecal expression characteristics of FA transport and transfer genes was distinct (Table 4). Cecal expression of *Abcc2, Dhfr, Folr, Pcft*, and *Rfc* was lower than that in the other parts of the intestines, whereas that of *Abcc3* was higher, and they were not different among groups (*P* > 0.05), indicating differences in FA absorption efficiency between the intestinal segments, and that the contribution of folate absorption in cecum may be relatively small.

**Table 4:** Relative mRNA expression of genes involved in folic acid absorption in the intestines.

**Items**		**FA0**	**FA1**	**FA6**	**FA24**	**SEM**	***P*-value**
*Abcc2*	Duodenum	1.007	0.857	0.812	1.269	0.088	0.267
	Jejunum	1.215	0.969	0.826	1.096	0.117	0.735
	Ileum	2.412^Aa^	1.077^Bb^	0.658^Bb^	0.819^Bb^	0.231	0.003
	Cecum	0.007	0.014	0.006	0.003	0.002	0.340
*Abcc3*	Duodenum	1.008	2.483	2.777	2.452	0.307	0.161
	Jejunum	1.817^AaB^	0.935^Aa^	3.033^Bb^	1.819^AaB^	0.260	0.009
	Ileum	3.865^b^	1.174^a^	2.952^b^	2.689^b^	0.346	0.015
	Cecum	7.551	6.498	6.815	7.397	0.676	0.958
*Abcc5*	Duodenum	1.137	1.062	1.179	0.839	0.115	0.787
	Jejunum	0.961	0.721	1.137	0.798	0.087	0.372
	Ileum	0.952	0.693	0.963	0.592	0.119	0.672
	Cecum	0.651	0.915	1.037	0.529	0.092	0.177
*Pcft*	Duodenum	1.022	0.625	0.639	0.761	0.063	0.067
	Jejunum	0.471^a^	0.229^b^	0.294^ab^	0.315^ab^	0.046	0.049
	Ileum	0.604	0.623	0.673	0.384	0.058	0.335
	Cecum	0.128	0.091	0.121	0.100	0.011	0.645
*Rfc*	Duodenum	1.007^a^	1.564^b^	0.564^a^	0.942^a^	0.127	0.015
	Jejunum	1.208^a^	0.559^b^	0.680^b^	0.966^ab^	0.093	0.026
	Ileum	1.271^a^	0.830^ab^	0.576^b^	0.906^ab^	0.094	0.035
	Cecum	0.506	0.647	0.507	0.458	0.046	0.557
Folr	Duodenum	1.002^Aa^	0.371^Cc^	0.219^Cc^	0.550^Bb^	0.090	<0.001
	Jejunum	0.341	0.271	0.119	0.288	0.037	0.179
	Ileum	0.792^a^	0.291^b^	0.461^b^	0.343^b^	0.072	0.025
	Cecum	0.014	0.020	0.013	0.012	0.002	0.508
*Dhfr*	Duodenum	1.007^Aa^	0.947^Aa^	0.514^Bb^	0.517^Bb^	0.077	0.002
	Jejunum	0.526	0.369	0.578	1.036	0.102	0.087
	Ileum	0.722^Aa^	0.410^Bb^	0.256^Bc^	0.386^Bbc^	0.055	<0.001
	Cecum	0.231	0.391	0.494	0.332	0.040	0.099

*Data are expressed as mean and pooled standard error of the mean (SEM) (n = 6). Mean values within a row with different lowercase superscript letters are significantly different at P < 0.05, and mean values with different uppercase superscript letters are significantly different at P < 0.01. FA, folic acid supplementation. The numbers after FA refer to the amounts added in mg/kg feed. Abcc, ATP-binding cassette subfamily C member; Dhfr, dihydrofolate reductase; Folr, folate receptor; Pcft, proton coupled folate transporter; Rfc, reduced folate carrier*.

*Pcft, Rfc*, and *Folr* are relevant in FA absorption from the intestinal lumen into enterocytes. Duodenal *Pcft* expression showed a decreasing tendency (*P* = 0.067), whereas jejunal *Pcft* expression decreased (*P* = 0.049) in treatment groups. Duodenal *Rfc* expression was higher (*P* = 0.015) in birds in the FA1 group compared to birds in the other treatment groups, while jejunal *Rfc* expression was lower in birds in the FA1 and FA6 group (*P* = 0.026) and ileal *Rfc* expression was significantly lower in birds in the FA6 group (*P* = 0.035) compared to birds in control group. Duodenal and ileal *Folr* expression significantly decreased (*P* < 0.001, and *P* = 0.025, respectively) in birds in the treatment groups compared with that of birds in the control groups. The down regulation of these genes may suggest a decrease in FA transport from the intestinal lumen into the enterocytes.

When FA is absorbed into enterocytes, it must be converted into its active biological form, tetrahydrofolate, by DHFR. Duodenal *Dhfr* expression decreased in birds in the FA6 and FA24 treatment groups compared to birds in the control and FA1 groups (*P* = 0.002); jejunal *Dhfr* expression in the laying hens decreased in the treatment groups (*P* < 0.001). The absorption of FA may be limited by the down-regulation of *Dhfr* due to that FA cannot absorption into blood directly and must be reduced into THF by DHFR.

*Abcc3* and *Abcc5* are involved in the process of expelling folate from enterocytes into the vascular system, and *Abcc2* is involved in the process of pumping folates back into the intestinal lumen. The *Abcc5* expression in the different intestinal segments was not affected by dietary FA supplementation. Jejunal and ileal *Abcc3* expression first decreased in FA1 group and then rose in FA6 group (*P* = 0.009, and *P* = 0.015, respectively). Ileal *Abcc2* expression of the laying hens decreased in treatment groups (*P* = 0.003). That *Abcc2* and *Abcc3* expression changes with the concentration of FA in diet implies their involvement in the maintenance of folate homeostasis in the intestinal epithelium.

### Effects of Folic Acid Supplementation on Cecal Microbiota of Laying Hens

**Figure 1:**
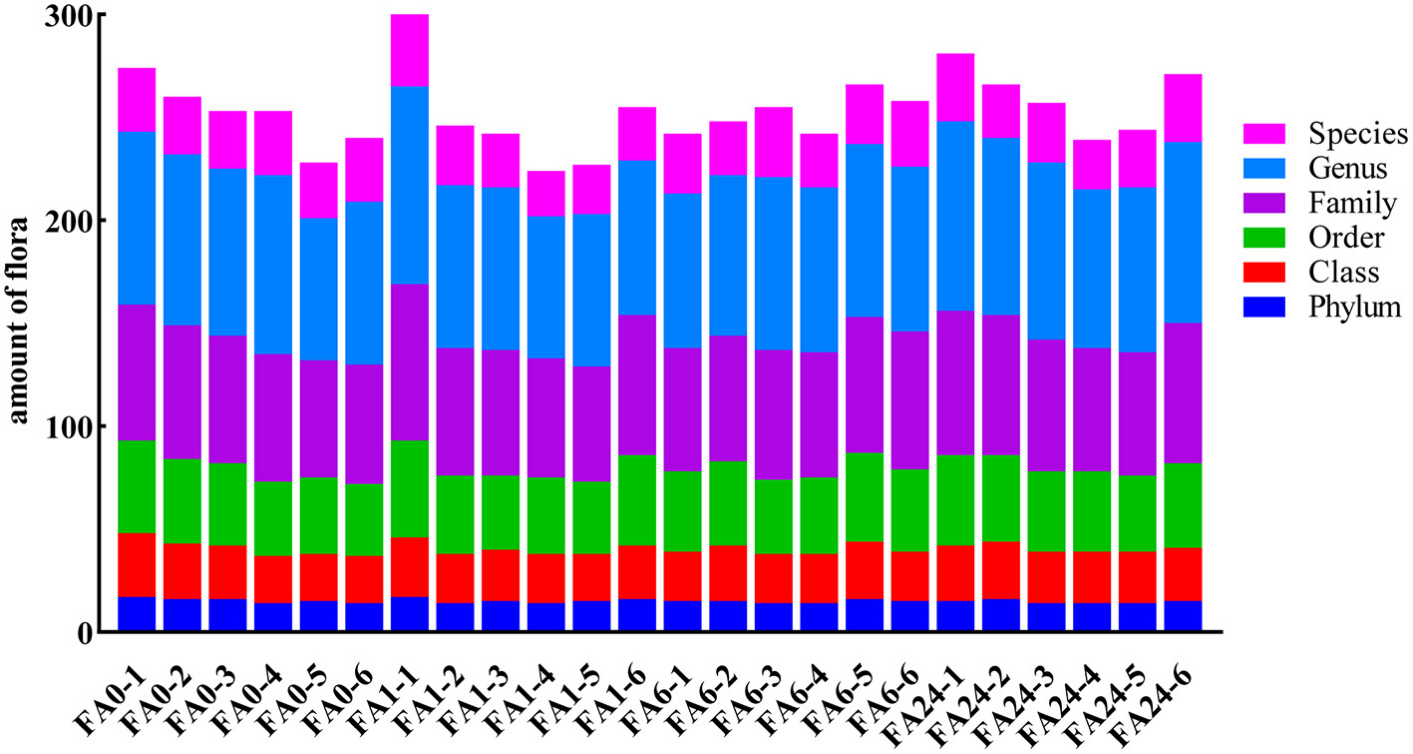
The composition of the cecal microbiota at different classification levels. The taxonomic-composition distribution histograms of each sample are shown at the phylum, class, order, family, genus, and species levels separately. FA, folic acid supplement, the numbers after FA refer to the amounts added in mg/kg feed, the numbers after (-) indicate samples in each group.

A total of 1,243,900 sequence tags with a median length of 425 bp (V3 + V4, ~402 to 453 bp) were obtained from the samples after removing redundant sequences. Each sample from the experimental laying hens contained 52,794 ± 5,832 sequence tags. The sequences were further clustered into 35,392 OTUs using a 97% similarity cut-off value. Rarefaction curves generated from the OTUs suggested that a high sampling coverage (~99%) was achieved for all samples. Bacterial community richness index values (Chao1 and ACE) and diversity index values (Shannon and Simpson) were not affected (*P* > 0.05) by FA supplementation (Table 5). Based on unweighted UniFrac distance analysis, the β-diversity of cecal microbiota was significantly affected by the treatments (*R* = 0.2312; *P* = 0.003); however, analysis based on weighted UniFrac distance showed that the β-diversity of the cecal microbiota was not affected (*R* = 0.0076; *P* = 0.375) by dietary FA supplementation. Furthermore, according to the OTU classification and taxonomic position identification, the cecal microbial composition of the hens was not affected by dietary FA supplementation at the phylum, class, order, family, genus, and species levels (Figure 1).

**Table 5:** Effect of folic acid on the cecal microbial diversity in laying hens.

**Items**	**FA0**	**FA1**	**FA6**	**FA24**	**SEM**	***P*-value**
Simpson	0.9910	0.9907	0.9902	0.9913	0.0005	0.890
Chao1	1969.6	1839.4	1919.4	1968.1	65.68	0.899
ACE	2032.7	1926.2	1993.8	2073.6	68.98	0.906
Shannon	8.33	8.32	8.33	8.41	0.60	0.960

*Data are expressed as the means and pooled standard error of the mean (SEM) (n = 6). FA, folic acid supplement, the numbers after FA refer to the amounts added in mg/kg feed; ACE, abundance-based coverage estimator metric; Chao1, Chao1 richness estimator; Shannon, Shannon diversity index; Simpson, Simpson index*.

**Figure 2:**
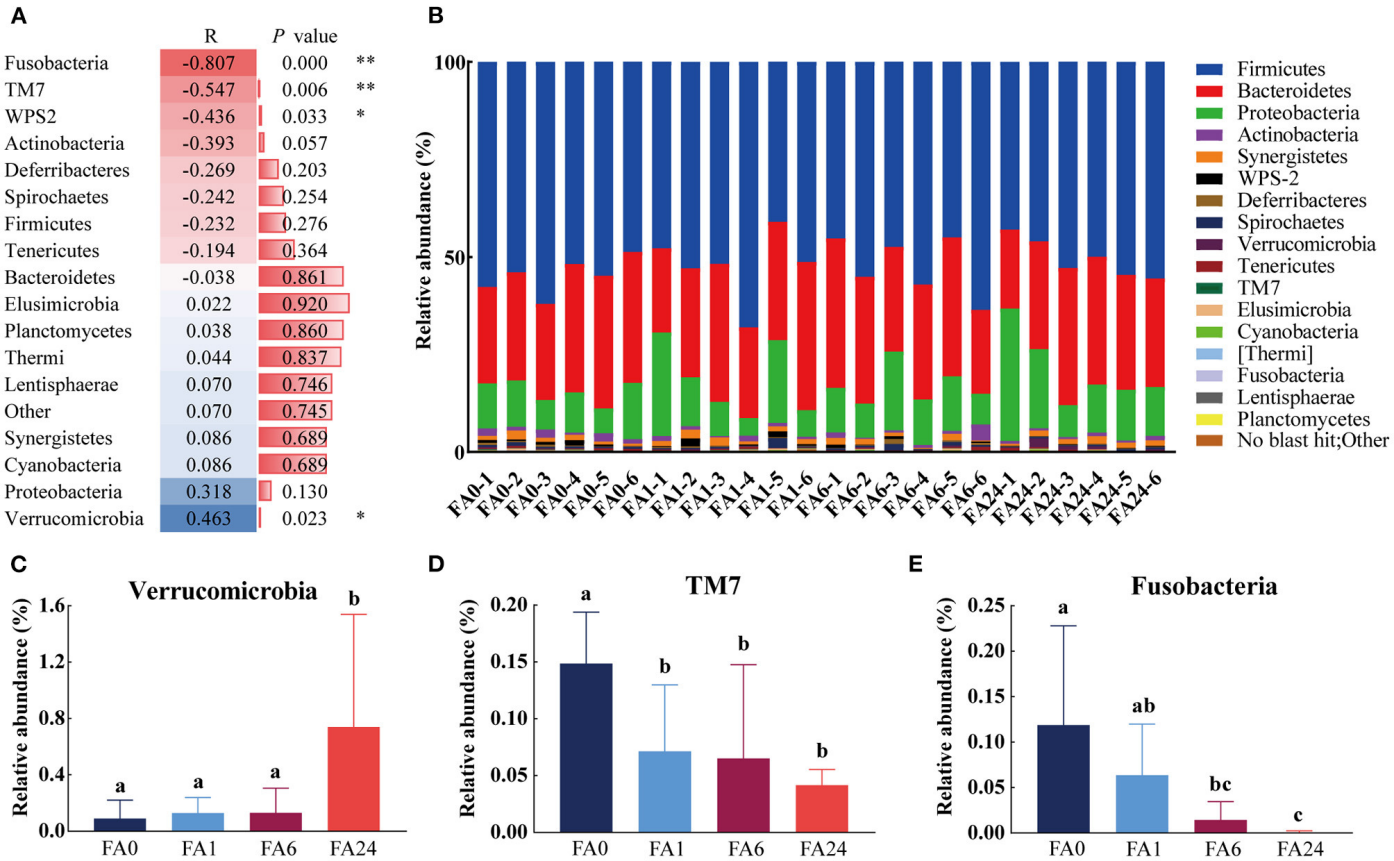
Distribution of the cecal microbiota at the phylum level. **(A)** Spearman's correlation coefficients of dietary folic acid supplementation and the relative abundance of bacteria at the phylum level, ** indicated highly significant correlation (*P* < 0.01), * indicated significant correlation (*P* < 0.05). **(B)** Taxonomic composition at the phylum level across different samples. **(C)–(E)** Comparison of the relative abundance of Verrucomicrobia,TM7, and Fusobacteria in different groups, respectively. Different letters in the column represent significant differences (*P* < 0.05). FA, folic acid supplement, the numbers after FA refer to the amounts added in mg/kg feed, the numbers after (-) indicate samples in each group.

A total of 17 phyla were identified, with Firmicutes, Bacteroidetes, and Proteobacteria accounting for almost 95% of the sequences. Firmicutes was the most abundant phylum (45 to 68%) in all four groups, followed by Bacteroidetes (21 to 38%) and Proteobacteria (4 to 26%). The remaining 14 phyla accounted for only 5% of the total. The relative abundance of Verrucomicrobia, TM7, and Fusobacteria were affected (*P* < 0.05) by dietary FA supplementation. Higher FA consumption seemed to be associated with lower relative abundance of Fusobacteria, TM7, and WPS-2, and a higher relative abundance of Verrucomicrobia (Figure 2).

**Figure 3:**
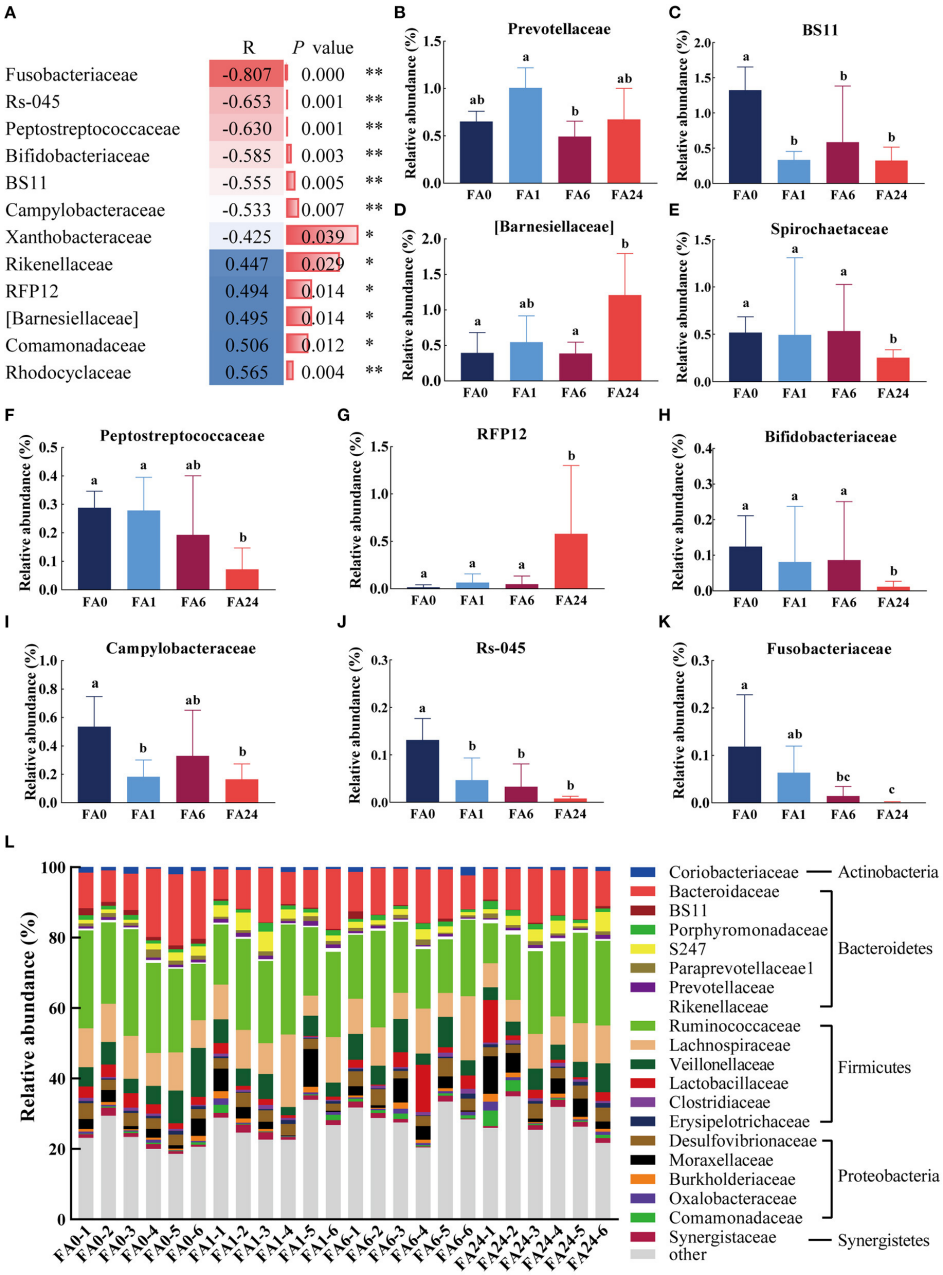
Distribution of the cecal microbiota at the family level. **(A)** Spearman's correlation coefficients of dietary folic acid supplementation and the relative abundance of bacteria at the family level [only statistically significant differences (*P* < 0.05) are shown], ** indicated highly significant correlation (*P* < 0.01), * indicated significant correlation (*P* < 0.05). **(B–K)** Comparison of the relative abundance of Prevotellaceae, BS11, [Barnesiellaceae], Spirochaetaceae, Peptostreptococcaceae, RFP12, Bifidobacteriaceae, Campylobacteraceae, Rs-045, Fusobacteriaceae, respectively. Different letters in the column represent significant differences (*P* < 0.05). **(L)** Taxonomic composition at the family level across different samples (top 20 classified bacteria are shown). FA, folic acid supplement, the numbers after FA refer to the added in mg/kg feed, the numbers after (-) indicate samples in each group.

At the family level, 107 families were identified in the cecal contents, of which, 71 families each accounted for more than 0.1% of the total microbial population in at least one sample and were selected for further analysis. After elimination of unclassified families, there were significant differences in the relative abundance of 10 families in the cecum of the laying hens. Spearman correlation coefficient analysis showed that the relative abundance of seven families was negatively correlated with dietary FA supplementation, whereas the relative abundance of five families was positively correlated with dietary FA supplementation (Figure 3).

**Figure 4:**
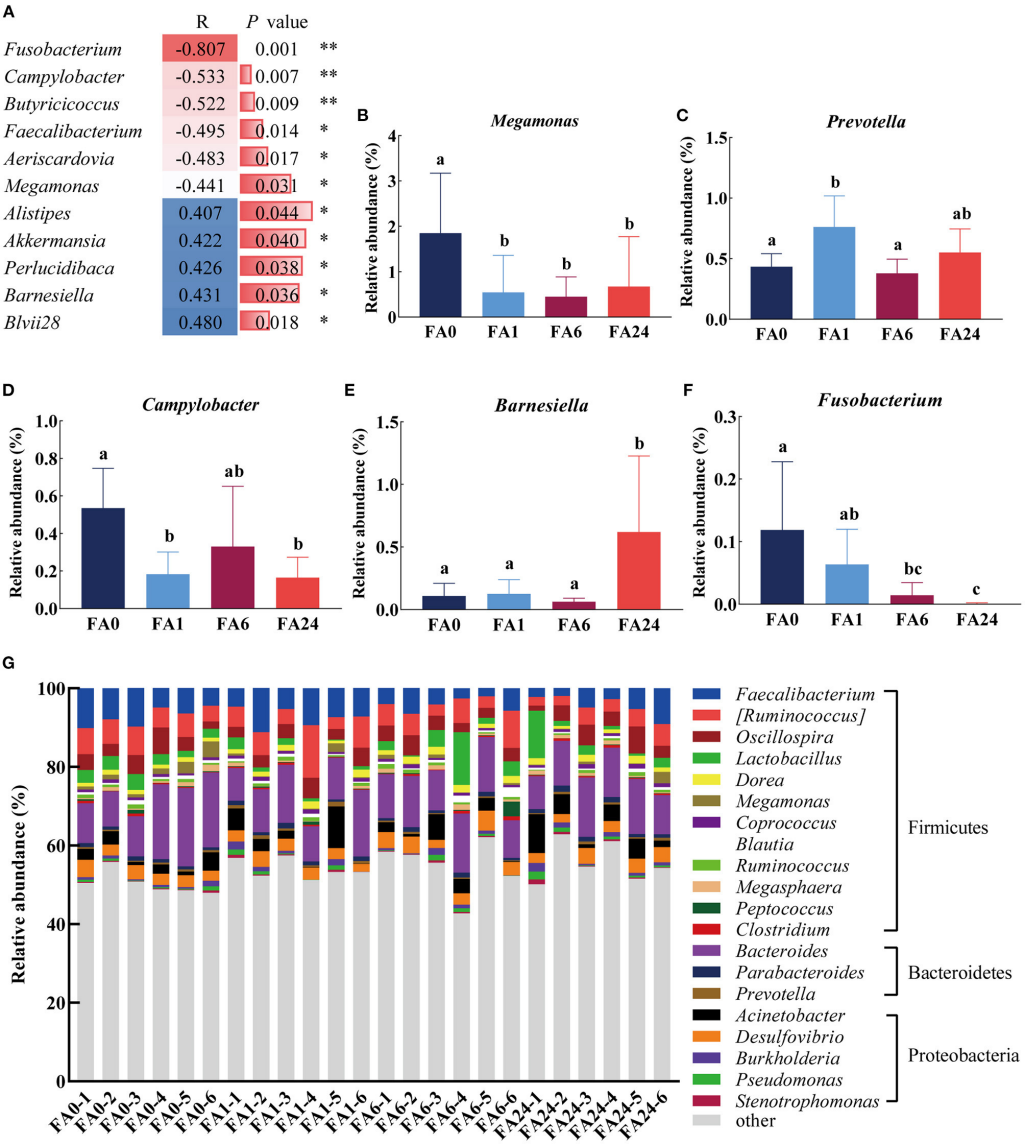
Distribution of the cecal microbiota at the genus level. **(A)** Spearman's correlation coefficients of dietary folic acid supplementation and the relative abundance of bavteria at the genus level [only statistically significant differences (*P* < 0.05) are shown], ** indicated highly significant correlation (*P* < 0.01), * indicated significant correlation (*P* < 0.05). **(B–F)** Comparison of the relative abundance of *Megamonas, Prevotella, Campylobacter, Barnesiella, Fusobacterium*, respectively. Different letters in the column represent significant differences (*P* < 0.05). **(G)** Taxonomic composition at the genus level across different samples. (top 20 classified bacteria are shown). FA, folic acid supplement, the numbers after FA refer to the amounts added in mg/kg feed, the numbers after (-) indicate samples in each group.

At the genus level, 193 genera were identified in the cecal contents, of which, 105 genera each accounted for more than 0.1% of the population in at least one sample and were selected for further analysis. After eliminating unclassified genera, we observed that there were significant differences in the relative abundances of five genera in the cecum of the laying hens (*P* < 0.05). Spearman correlation coefficient analysis showed that the relative abundance of six genera was negatively correlated with dietary FA supplementation, whereas the relative abundance of five genera was positively correlated with dietary FA supplementation (Figure 4). Fusobacteriaceae and *Fusobacterium* were the only family and genus classified in Fusobacteria, and *Campylobacter* was the only genus classified in Campylobacteraceae. Therefore, only *Fusobacterium* and *Campylobacter* are discussed in the following sections.

In summary, compared with the FA0 group, the number of different bacteria in the FA1, FA6, and FA24 groups were 7, 7, and 16 at the three levels, respectively, and 4, 8, and 6 bacteria differed between FA1 and FA6, FA1 and FA24, FA6 and FA24, respectively (Appendix Table 2).

The functional profiles of the microbiota based on the analysis of KEGG level 2 categories showed six pathways were different among the relative abundance of the groups (*P* < 0.05). Four pathways were positively correlated with the dietary FA supplementation, whereas seven pathways were negatively correlated with the dietary FA supplementation. Of the pathways, four (membrane transport, carbohydrate metabolism, metabolism of cofactors and vitamins, and nucleotide metabolism) were among the top 10 according to relative abundance. The number of different predominant gene functions of the three pair-wise comparisons of FA0, FA1, and FA6 was one. Compared with FA24, the numbers of different predominant gene functions of FA0, FA1, and FA6 groups were 10, 6, and 5, respectively. The microbial gene functions related to metabolic pathways (carbohydrate metabolism, metabolism of cofactors and vitamins, nucleotide metabolism, and metabolic diseases) were negatively correlated with dietary FA supplementation (Table 6).

**Table 6:** Main microbial pathways grouped into level-2 KEGG functional categories using PICRUSt.

**Item**	**FA0**	**FA1**	**FA6**	**FA24**	**non-parametric test *P* value**	**Spearman correlation R**	**Correlation *P* value**	**FA0 vs. FA1**	**FA0 vs. FA6**	**FA0 vs. FA24**	**FA1 vs. FA6**	**FA1 vs. FA24**	**FA6 vs. FA24**
Cell growth and death	0.552	0.551	0.550	0.538	0.012	−0.603	0.002			down		down	down
Cell motility	2.141	2.229	2.240	2.519	0.061	0.506	0.012			up		up	up
Unclassified; cellular processes and signaling	4.093	4.149	4.091	4.131	0.026	0.226	0.288	up		up	down		
Membrane transport	12.4	12.433	12.539	12.689	0.092	0.506	0.012			up		up	
Signal transduction	1.594	1.655	1.664	1.790	0.039	0.549	0.005			up		up	up
Metabolic diseases	0.096	0.093	0.096	0.091	0.093	−0.425	0.038			down			
Folding, sorting, and degradation	2.446	2.428	2.418	2.372	0.007	−0.721	<0.001		down	down		down	
Unclassified; genetic information processing	2.681	2.664	2.663	2.631	0.160	−0.474	0.019						
Carbohydrate metabolism	10.824	10.727	10.704	10.517	0.220	−0.415	0.044						
Metabolism of cofactors and vitamins	4.43	4.414	4.387	4.302	0.040	−0.555	0.005			down		down	down
Nucleotide metabolism	3.952	3.862	3.907	3.793	0.093	−0.409	0.047			down			down
Unclassified; metabolism	2.386	2.415	2.41	2.458	0.042	0.56	0.004			up			
Total								1	1	10	1	6	5

*Data are expressed as the means of relative abundance (%) (n = 6). FA, folic acid supplement, the numbers after FA refer to the amounts added in mg/kg feed*.

**Figure 5:**
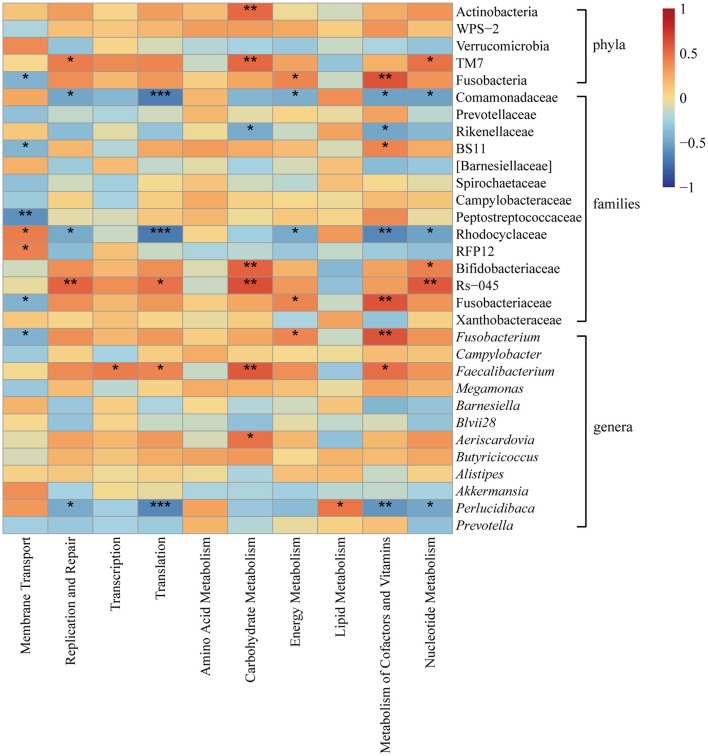
Heatmap illustrating correlation between the predicted gene functions (the KEGG level 2 categories in the top 10) and bacteria with different relative abundances among groups at the phylum, family and genus levels. The color scale indicates the degree of correlation (red: strong positive correlation, blue: strong negative correlation). *P < 0.05, **P < 0.01, ***P < 0.001.

The correlation between the bacteria of different relative abundance and the top 10 microbial gene functions was analyzed (Figure 5). At the phylum level, Actinobacteria and TM7 were positively correlated with carbohydrate metabolism, and TM7 was positively correlated with nucleotide metabolism. At the family level, the Rs-045 group in TM7 and Bifidobacteriaceae were positively correlated with carbohydrate metabolism and nucleotide metabolism; the BS11 group in Bacteroidetes was positively correlated with the metabolism of cofactors and vitamins, while Comamonadaceae and Rhodocyclaceae were negatively correlated with functions involved in the metabolism of cofactors, and vitamins and nucleotide metabolism; Rikenellaceae was negatively correlated with carbohydrate metabolism, and the metabolism of cofactors and vitamins. The RFP12 group in Verrucomicrobia and Rhodocyclaceae were positively correlated with membrane transport, while BS11 and Peptostreptococcaceae were negatively correlated with membrane transport. At the genus level, *Fusobacteria* was positively correlated with the metabolism of cofactors and vitamins, and negatively correlated with membrane transport. *Faecalibacterium* and *Aeriscardovia* were positively correlated with carbohydrate metabolism; additionally, *Faecalibacterium* was positively correlated with the metabolism of cofactors and vitamins. *Perlucidibaca* was negatively correlated with the metabolism of cofactors and vitamins, and nucleotide metabolism.

## Discussion

### Folic Acid Absorption Characteristic in the Intestines of Laying Hens

Plasma folate concentration appeared to reach saturation at a FA concentration between 2 and 4 mg/kg of the hen diet (16). In the present study, the serum folate level reached a plateau at a dietary FA concentration of 6 mg/kg. Perhaps, the strain used in this study had a different sensitivity to FA. The sensitivity of plasma homocysteine to folate status is well-documented the literature, which can be attributed to the role of folate in homocysteine re-methylation to methionine (18). Tactacan et al. (36) reported that the inclusion of crystalline FA or 5-methyltetrahydrofolic acid (5-MTHF) in the hens' diet increased serum and egg folate levels and decreased plasma homocysteine level. Although the effect of FA supplementation on plasma homocysteine level may differ between hen strains, the decrease in homocysteine level is not indefinite and it reaches a plateau, similar to that of serum folate (16). These findings indicate that FA absorption in the intestines, metabolism in the liver, and excretion into the blood is well-regulated. The intestines, liver, and kidneys play essential roles in regulating folate homeostasis in the body.

The mucosal-to-serosal FA uptake rate in the jejunum of laying hens reaches a plateau at FA concentrations higher than 0.1 μM; this pattern is reminiscent of the response demonstrated by laying hens in egg folate concentration when their dietary FA supplementation is increased (24). We assume that a folate homeostasis regulation mechanism exists in intestinal epithelial cells. In the present study, we focused on intestinal factors that might play a role in folate homeostasis. The relative mRNA expression levels of transporters and enzymes involved in folate homeostasis in the duodenum, jejunum, ileum, and cecum of laying hens were compared.

The mRNA expression of enzymes and transporters involved in FA absorption was detected in the four segments of the intestinal tract, indicating the presence of an FA transport system throughout the intestinal tract of laying hens. However, the uptake rate was higher in the duodenum and jejunum than in the ileum and cecum (24). *Rfc* and *Pcft* were extensively expressed in most tissues, suggesting that they play important roles in chickens. FA supplementation (10 mg/kg) did not affect the expression of these genes, but jejunal *Rfc* expression exhibited a decreasing trend with the increase in dietary FA supplementation (21, 22). In the present study, there was no change in *Pcft* expression levels in the experimental groups, although a tendency for reduced *Pcft* expression was observed, which concurs with the findings of Jing et al. (22). There was a significant increase in the duodenal *Rfc* expression levels and a significant decrease in the jejunal *Rfc* expression levels of hens in the FA1 group compared with those of hens in the other treatment groups, indicating that *Rfc* expression might be regulated by FA concentration in the intestines. Folate oversupplementation led to down-regulation of intestinal folate uptake in rat, which was caused by down-regulating the expressions of RFC and PCFT in the protein level (37). Under the folate oversupplemented condition, the uptake of folic acid by Caco-2 and HK-2 cell was specifically lower than the folate-sufficient condition, which was associated with the decrease in mRNA levels of *RFC, PCFT*, and *FOLR*, and also the decrease in protein level of RFC and the activity of the RFC promoter (38). We speculate that, *Rfc, Pcft*, and *Folr* transcription processes and their protein levels may also be regulated in chicken intestine, and the down-regulation of these genes would contribute to a lower folic acid absorption efficiency.

Although apical uptake transporters have been extensively explored, the molecular entities involved in basolateral efflux transport and reverse transport to the lumen are yet to be identified in chickens. ABCC3 is involved in the serosal efflux of FA and leucovorin, as well as 5-MTHF in mice (39), and the down- and up-regulation of ABCC3 expression can influence 2,008 human ovarian cells' folate homeostasis (40). Although duodenal *Abcc3* expression was not different between treatment groups, it showed an increasing trend with the increase in dietary FA supplementation, indicating that folate transfer from the intestinal epithelial cells into the bloodstream might be regulated to maintain body folate homeostasis. Jejunal and ileal *Abcc3* expression decreased in the FA1 group and then increased in the FA6 and FA24 groups, which may indicate that priority may be given to meet body need when the diet folate is short. Efflux may increase to maintain folate homeostasis when the diet folate is in excess. Ileal *Abcc2* expression decreased in treatment groups indicating that there was a balance of folate concentration between intestinal lumen and epithelial cells. Further studies should pursue this.

The DHFR-mediated dihydrofolate reduction can be inhibited by its own substrate folic acid (41) and its production (6s)-5,6,7,8-tetrahydrofolate (42). Duodenal DHFR activity was reported to be lower in birds fed diets supplemented with 10 mg/kg FA than in birds in the control group (36). In the present study, duodenal and ileal *Dhfr* expression decreased with the increase in dietary FA supplementation, which might be associated with a decrease in DHFR activity.

In summary, FA transport and transfer from the intestinal lumen into enterocytes, and then into the bloodstream, are strictly regulated. It can be concluded that feeding excessively high levels of FA can cause large amounts of unabsorbed FA or its degradants to flow into the cecum of laying hens.

### Effect of Folic Acid Supplementation on the Cecum Chyme Microbiota in Laying Hens

Cecal microbiota plays an important role in chicken health, growth performance (43), and egg production (44). Although unabsorbed FA or its degradants flowed into the cecum in the experimental groups, our data suggest that there was no change in the microbial diversity due to FA supplementation. This outcome might be associated with the minor effect of FA supplementation on egg production performance (Appendix Table 1).

Excess consumption of folate by humans can significantly increase the relative abundance of *Faecalibacterium, Subdoligranulum, Alistipes, Haemophilus, Desulfovibrio, Prevotella, Odoribacterium, Dialister*, and *Akkermansia*, and significantly decrease the relative abundance of *Lachnospiraceae*, and *Erysipelatoclostridium* at the genus level (45). In the present study, the relative abundance of *Alistipes, Akkermansia, Barnesiella, Perlucidibaca*, and *Blvii28* were positively correlated with FA supplementation, whereas the relative abundance of *Faecalibacterium, Fusobacterium, Campylobacter, Butyricicoccus, Aeriscardovia*, and *Megamonas* were negatively correlated. The abundance of *Prevotella* exhibited a quadratic pattern, peaking at 1 mg/kg diet (FA1 group) and decreasing at higher supplementation levels (FA6 and FA24 groups). These discrepancies might be attributed to differences in intestinal microbiota between humans and chickens. Additionally, *Escherichia coli* and *Salmonella* are major pathogen in poultry industry (46–48), the biochemical modulation of folate may potentially help the host cope with those challenging situations (49) and increase the host survival (50). Nevertheless, they were not detected by 16S sequencing in the present study, and a further study is required.

Compared with FA0, the relative abundance of TM7, Rs-045 groups in TM7, BS11 groups in Bacteroidetes, *Campylobacter, Megamonas*, and *Fusobacterium* were decreased in the cecum of hens fed FA-supplemented diets, which might indicate that these bacterial classes were sensitivity to FA. TM7 is associated with oral inflammation, and its abundance increases in the patient's mouth with gingivitis severity and periodontal disease (51). *Fusobacterium* is a proinflammatory bacterium. In colorectal cancer tumors, *Fusobacterium* species are over-represented and a significant co-occurrence was observed for *Fusobacterium, Leptotrichia*, and *Campylobacter* species, which was associated with the Interleukin-8 gene and over-expression of other host genes (52). There was a high *Fusobacterium* abundance in 53-week-old hens with poor egg production, indicating the negative effects of *Fusobacterium* on chicken health (53). *Campylobacter spp*. are associated with the gastrointestinal tract of several animals, and *Campylobacter jejuni* and *C. coli* cause ~90% of human campylobacteriosis cases (foodborne bacterial gastroenteritis causing bloody diarrhea, fever, and abdominal pain). Chickens are natural hosts for *Campylobacter*, and chicken meat and eggs are the most important sources of human campylobacteriosis (54). *Campylobacter* is a commensal microorganism without any obvious clinical signs; however, oral uptake of *C. jejuni* can significantly affect nutrient absorption, intestinal epithelial barrier activity, and immune reaction, resulting in decreased weight gain in seemingly healthy chickens (55). The BS11 groups in Bacteroidetes participates in multiple pathways for fermenting hemicellulose monomeric sugars to short-chain fatty acids (56). *Megamonas* species are important propionate and acetate producers (57). In the present study, we found that BS11 and *Megamonas* were positively but weakly correlated with carbohydrate metabolism. Briefly, dietary FA supplementation can decrease the number of pathogenic bacteria in the intestines and the number of genera associated with short-chain fatty acid production.

There was a significant increase in the relative abundances of Verrucomicrobia, RFP12 family (within Verrucomicrobia), and Barnesiella (within Bacteroidetes), and a significant decrease in the relative abundance of Peptostreptococcaceae (within Firmicutes), Bifidobacteriaceae (within Actinobacteria), and Spirochaetaceae (within Spirochaetes) in the intestines of birds in the FA24 group compared to birds in the other treatment groups. The relative abundance of *Akkermansia* accounted for the highest proportion in Verrucomicrobia, and it was positively correlated with dietary FA supplementation. Zhang et al. (58) reported that chemical folate modulated the microbiota of rats by increasing the abundance of the genus *Akkermansia*, which further increased the relative abundance of the phylum Verrucomicrobia to the second most abundant phylum. Verrucomicrobia can improve glucose metabolism in animals (59), and have the potential to induce regulatory immunity (60). *Akkermansia* is an important microorganism because of its probiotic role, including thickening of the mucous layer, improving gut barrier function (61), enhancing glucose tolerance, reducing insulin resistance, modulating pathways involved in establishing homeostasis for basal metabolism, enhancing immune tolerance against commensal microbiota (62), and prolonging the life of mice from premature aging (63). Barnesiella is an effective immunomodulator that inhibits the colonization of pathogenic antibiotic-resistant bacteria in the gut (64), and promote the nutrient metabolism of chicken (65). Characterized Peptostreptococcaceae species are anaerobic bacteria that include pathogens associated with tissue infections and antibiotic resistance (66). Bifidobacteriaceae are considered to be the most important beneficial microbes in the gut (67), and some species in genus *Bifidobacterium* have the capacity for *de novo* folate biosynthesis, and are developed into probiotic products (68). FA supplementation of the laying hen diet at 24 mg/kg increased the relative abundance of beneficial bacteria and decreased the relative abundance of pathogenic bacteria, which were involved in the regulation of immunity. However, there was a decrease in the relative abundance of Bifidobacteriaceae, a beneficial bacterium, in the intestines of birds in the FA24 treatment group, this may suggest the folic acid supplementation associate with probiotics-Bifidobacteria The gene functions of microbiota in the intestines of birds in the FA24 group were significantly different from those of birds in the other treatment groups, indicating that 24 mg/kg FA supplementation may influence folate metabolism in laying hens. In conclusion, FA supplementation at 24 mg/kg was not suitable for laying hens.

According to the findings of our previous work, a suitable amount of dietary FA for folate-enriched egg production is 6 mg/kg of the diet. In this study, compared with the FA1 group, there was a significant decrease in the relative abundance of Prevotellaceae and *Prevotella*, and gene function associated with unclassified cellular processes and signaling in the intestines of hens in the FA6 group. Prevotellaceae are known degraders of intestinal mucus and can destroy this important intestinal barrier (69). This may indicate that dietary FA supplementation has little effect on the health and metabolism of laying hens for folate-enriched egg production from the microbial perspective.

## Conclusions

Serum folate level of laying hens reached a plateau in the FA6 group, which was possibly associated with the regulation of the expression of genes involved in FA absorption in the intestine. Unabsorbed FA may enter the cecum when added in excess, leading to a decrease in the relative abundance of some pathogenic bacteria, especially in the FA24 group. The beneficial bacteria (Bifidobacteriaceae) decreased in FA24 group. Overall, FA supplementation at 6 mg/kg, which was selected for folate-enriched egg production, did not affect the health and metabolism of laying hens negatively.

## Data Availability Statement

The datasets presented in this study can be found in online repositories. The names of the repository/repositories and accession number(s) can be found below: http://www.ncbi.nlm.nih.gov/bioproject/747060.

## Ethics Statement

The animal study was reviewed and approved by Shanxi Agricultural University Animal Experiment Ethics Committee.

## Author Contributions

YY: conceptualization, resources, supervision, project administration, and funding acquisition. YB: methodology, formal analysis, and writing—original draft preparation. RRL: investigation. XTW: date curation. RW and YB: writing—reviewing and editing. All authors have read and agreed to published version of the manuscript.

## Conflict of Interest

The authors declare that the research was conducted in the absence of any commercial or financial relationships that could be construed as a potential conflict of interest.

## Publisher's Note

All claims expressed in this article are solely those of the authors and do not necessarily represent those of their affiliated organizations, or those of the publisher, the editors and the reviewers. Any product that may be evaluated in this article, or claim that may be made by its manufacturer, is not guaranteed or endorsed by the publisher.
