# Supplementary material for: Folic Acid Absorption Characteristics and Effect on Cecal Microbiota of Laying Hens
Source: Front Vet Sci. 2021 Aug 17;8:720851. doi: 10.3389/fvets.2021.720851 (PMC8416075; doi:10.3389/fvets.2021.720851)
Supplement: Supplementary file 1 [file Table_1.DOCX]

**Appendix Table A.1** Effect of folic acid on production performance ^a^ and egg quality ^b^ in laying hens

| Items | FA0 | FA1 | FA6 | FA24 | SEM | *P*-value |
| --- | --- | --- | --- | --- | --- | --- |
| Egg production (%) | 83.94 | 87.04 | 85.95 | 84.98 | 0.65 | 0.669 |
| Feed intake (g/hen per day) | 130.09 | 134.92 | 126.41 | 128.39 | 1.41 | 0.852 |
| Egg mass (g/hen per day) | 53.76 | 56.68 | 54.20 | 55.30 | 0.42 | 0.334 |
| Feed conversion (g of feed/g of egg) | 2.428 | 2.448 | 2.547 | 2.327 | 0.015 | 0.612 |
| egg weight (g) | 64.04 | 63.66 | 63.07 | 65.08 | 0.60 | 0.334 |
| Shape index | 1.274 | 1.274 | 1.276 | 1.264 | 0.008 | 0.904 |
| Shell thickness (mm) | 0.374 | 0.364 | 0.368 | 0.371 | 0.001 | 0.201 |
| Yolk ratio (%) | 24.64 | 24.69 | 25.10 | 24.77 | 0.19 | 0.739 |
| Egg white ratio (%) | 64.63 | 63.94 | 63.53 | 64.87 | 0.47 | 0.319 |
| Shell ratio (%) | 11.30 | 11.37 | 11.37 | 10.98 | 0.09 | 0.445 |
| Shell strength (kg/cm^2^) | 4.288 | 4.075 | 4.282 | 4.324 | 0.037 | 0.142 |
| Albumen height (mm) | 8.471 | 8.200 | 7.673 | 8.230 | 0.061 | 0.102 |
| Haugh unit | 89.25 | 90.39 | 86.37 | 86.47 | 0.72 | 0.097 |
| Yolk color | 5.949 | 5.595 | 5.571 | 5.846 | 0.058 | 0.438 |

Data are expressed as the means and pooled standard error of the mean (SEM) (n = 6 for egg production indicators, n = 24 for egg quality indicators). FA = folic acid supplement, the numbers after FA refer to the amounts added in mg/kg feed.

^a^ Daily egg production and egg weight were monitored during the trial, and the date was collected with a replicate as the experimental unit. Egg production was expressed as average hen-day production, calculated from total eggs divided by the total number of hen-days. Egg mass was also calculated as total egg weight divided by the total number of hen-days. Feed consumption was recorded on a replicate basis at weekly interval, and feed intake was calculated as total weight of feed consumption divided by the total number of hen-days. Feed conversion was expressed as g feed consumed/g egg produced.

^b^ Four eggs per replicate were randomly selected at the last day of every week, and measurements were performed on the day of collection. Egg weight, albumen height, Haugh units, and yolk color were assessed with an egg analyzer (EMT-5200; ORKA Food Technology Ltd., Ramat Hasharon, Israel). Eggshell strength was measured with an egg force reader (EFR-01; ORKA Food Technology Ltd.). Eggshell thickness was measured with an eggshell thickness gauge (Robotmation Co., Ltd., Tokyo, Japan) and is reported as the mean value of measurements at three locations on each egg (air cell, equator, and sharp end). The size of eggs was measured with a digital caliper (DeFeng measuring Tools Co., Ltd., Shenzhen, China), and the shape index was calculated according to the following formula: shape index = height/width. The yolk was weighed after removing the albumen, and the albumen mass was calculated as egg weight minus the yolk and shell weights. The percentage was calculated as a ratio of each to the egg weight. Date presented in this table were collected during the last week.
