# Supplementary material for: Folic Acid Absorption Characteristics and Effect on Cecal Microbiota of Laying Hens
Source: Front Vet Sci. 2021 Aug 17;8:720851. doi: 10.3389/fvets.2021.720851 (PMC8416075; doi:10.3389/fvets.2021.720851)
Supplement: Supplementary file 2 [file Table_2.DOCX]

**Appendix Table A.2** Effect of folic acid supplementation on cecal microbiota in laying hens

| Item | | FA0 | | FA1 | | FA6 | | FA24 | non-parametric test *P* value | Spearman correlation *R* | Correlation *P* value | FA0 vs FA1 | FA0 vs FA6 | FA0 vs FA24 | FA1 vs FA6 | FA1 vs FA24 | FA6 vs FA24 |
| --- | --- | --- | --- | --- | --- | --- | --- | --- | --- | --- | --- | --- | --- | --- | --- | --- | --- |
| p_Actinobacteria | | 1.484 | | 0.914 | | 1.330 | | 0.737 | 0.255 | -0.393 | 0.057 |  |  |  |  |  |  |
| p_Fusobacteria | | 0.118 | | 0.064 | | 0.014 | | 0.001 | 0.001 | -0.807 | ＜0.001 |  | down | down |  | down |  |
| p_TM7 | | 0.149 | | 0.071 | | 0.065 | | 0.042 | 0.039 | -0.547 | 0.006 | down | down | down |  |  |  |
| p_Verrucomicrobia | | 0.090 | | 0.129 | | 0.129 | | 0.739 | 0.049 | 0.463 | 0.023 |  |  | up |  |  | up |
| p_WPS-2 | | 0.613 | | 0.751 | | 0.353 | | 0.133 | 0.205 | -0.436 | 0.033 |  |  |  |  |  |  |
| p_Actinobacteria; f_Bifidobacteriaceae | | 0.125 | | 0.014 | | 0.086 | | 0.012 | 0.011 | -0.585 | 0.003 |  |  | down |  | down | down |
| p_Bacteroidetes; f_[Barnesiellaceae] | | 0.396 | | 0.548 | | 0.387 | | 1.207 | 0.046 | 0.495 | 0.014 |  |  | up |  |  | up |
| p_Bacteroidetes; f_BS11 | | 1.324 | | 0.335 | | 0.584 | | 0.326 | 0.016 | -0.555 | 0.005 | down | down | down |  |  |  |
| p_Bacteroidetes; f_Prevotellaceae | | 0.652 | | 1.007 | | 0.492 | | 0.674 | 0.027 | -0.172 | 0.421 |  |  |  | down |  |  |
| p_Bacteroidetes; f_Rikenellaceae | | 0.594 | | 0.597 | | 0.608 | | 0.939 | 0.101 | 0.447 | 0.029 |  |  |  |  |  |  |
| p_Firmicutes; f_Peptostreptococcaceae | | 0.288 | | 0.279 | | 0.193 | | 0.072 | 0.016 | -0.630 | 0.001 |  |  | down |  | down |  |
| p_Fusobacteria; f_Fusobacteriaceae | | 0.118 | | 0.064 | | 0.014 | | 0.001 | 0.001 | -0.807 | ＜0.001 |  | down | down |  | down |  |
| p_Proteobacteria; f_Xanthobacteraceae | | 0.043 | | 0.011 | | 0.025 | | 0.003 | 0.147 | -0.425 | 0.039 |  |  |  |  |  |  |
| p_Proteobacteria; f_Comamonadaceae | | 0.334 | | 0.786 | | 0.852 | | 1.622 | 0.070 | 0.506 | 0.012 |  |  |  |  |  |  |
| p_Proteobacteria; f_Rhodocyclaceae | | 0.065 | | 0.204 | | 0.123 | | 0.346 | 0.059 | 0.565 | 0.004 |  |  |  |  |  |  |
| p_Proteobacteria; f_Campylobacteraceae | | 0.535 | | 0.183 | | 0.331 | | 0.165 | 0.011 | -0.533 | 0.007 | down |  | down |  |  |  |
| p_Spirochaetes; f_Spirochaetaceae | | 0.518 | | 0.494 | | 0.536 | | 0.253 | 0.032 | -0.398 | 0.054 |  |  | down |  | down | down |
| p_TM7; f_Rs-045 | | 0.131 | | 0.047 | | 0.033 | | 0.008 | 0.01 | -0.653 | 0.001 | down | down | down |  |  |  |
| p_Verrucomicrobia; f_RFP12 | | 0.015 | | 0.066 | | 0.048 | | 0.58 | 0.033 | 0.494 | 0.014 |  |  | up |  | up | up |
| p_Actinobacteria; f_Bifidobacteriaceae; g_Aeriscardovia | | 0.103 | | 0.014 | | 0.085 | | 0.012 | 0.061 | -0.483 | 0.017 |  |  |  |  |  |  |
| p_Bacteroidetes; f_[Barnesiellaceae]; g_Barnesiella | | 0.107 | | 0.125 | | 0.062 | | 0.617 | 0.030 | 0.431 | 0.036 |  |  | up |  | up | up |
| p_Bacteroidetes; f_Prevotellaceae; g_Prevotella | | 0.433 | | 0.763 | | 0.379 | | 0.550 | 0.021 | 0.038 | 0.861 | up |  |  | down |  |  |
| p_Bacteroidetes; f_Rikenellaceae; g_Alistipes | | 0.077 | | 0.076 | | 0.079 | | 0.190 | 0.383 | 0.407 | 0.044 |  |  |  |  |  |  |
| p_Bacteroidetes; f_Rikenellaceae; g_Blvii28 | | 0.042 | | 0.120 | | 0.129 | | 0.156 | 0.075 | 0.480 | 0.018 |  |  |  |  |  |  |
| p_Firmicutes; f_Ruminococcaceae; g_Butyricicoccus | | 0.158 | | 0.071 | | 0.090 | | 0.035 | 0.076 | -0.522 | 0.009 |  |  |  |  |  |  |
| p_Firmicutes; f_Ruminococcaceae; g_Faecalibacterium | | 7.317 | | 7.556 | | 4.218 | | 4.405 | 0.052 | -0.495 | 0.014 |  |  |  |  |  |  |
| p_Firmicutes; f_Veillonellaceae; g_Megamonas | | 1.845 | | 0.546 | | 0.448 | | 0.672 | 0.049 | -0.441 | 0.031 | down | down | down |  |  |  |
| p_Fusobacteria; f_Fusobacteriaceae; g_Fusobacterium | | 0.118 | | 0.064 | | 0.014 | | 0.001 | 0.001 | -0.807 | ＜0.001 |  | down | down |  | down |  |
| p_Proteobacteria; f_Campylobacteraceae; g_Campylobacter | | 0.535 | | 0.183 | | 0.331 | | 0.165 | 0.011 | -0.533 | 0.007 | down |  | down |  |  |  |
| p_Proteobacteria; f_Moraxellaceae; g_Perlucidibaca | | 0.025 | | 0.091 | | 0.041 | | 0.123 | 0.221 | 0.426 | 0.038 |  |  |  |  |  |  |
| p_Verrucomicrobia; f_Verrucomicrobiaceae; g_Akkermansia | | 0.070 | | 0.053 | | 0.064 | | 0.149 | 0.113 | 0.422 | 0.040 |  |  |  |  |  |  |
| Total |  | |  | |  | |  | |  |  |  | 7 | 7 | 16 | 4 | 8 | 6 |

Data are expressed as the means of relative abundance (%) (*n*=6). FA = folic acid supplement, the numbers after FA refer to the amounts added in mg/kg feed. p = phylum; f = family; g = genus
